# Remineralization of early caries lesions by calcium hypophosphite in vitro: a surface microhardness study

**DOI:** 10.1038/s41405-026-00440-1

**Published:** 2026-05-08

**Authors:** Bennett Tochukwu Amaechi, Razina Vohra, Sima Abdollahi, Kelly Yang, Amos Chinedu Obiefuna, Erik Schulze zur Wiesche, Joachim Enax

**Affiliations:** 1 Department of Comprehensive Dentistry, School of Dentistry, University of Texas Health San Antonio, San Antonio, TX USA; 2 Department of Mathematics and Statistics, University of Maryland Global Campus, San Antonio, TX USA; 3 Research Department Dr. Kurt Wolff GmbH & Co. KG, Bielefeld, Germany

**Keywords:** Oral diseases, Dentistry

## Abstract

**Aim:**

This in vitro study used a pH cycling model to compare the caries remineralizing efficacy of toothpaste formulations containing calcium hypophosphite (CaP), hydroxyapatite (HAP), or sodium fluoride (NaF).

**Methods:**

Bovine enamel blocks with plaque-induced initial caries lesions were randomized (*n* = 30/group) to toothpaste formulations containing 1% CaP, 20% HAP, 1% CaP+20% HAP, or 1450 ppm fluoride provided as NaF. Lesion-bearing samples were subjected to a 14-day remineralization using a pH-cycling model with daily regimen of three 2-min applications of toothpaste slurry (1:3 toothpaste: water), one 2-h acid exposure, and storage in artificial saliva for the rest of the day. Remineralization was quantified as change in surface microhardness (SMH) of each sample measured before and after toothpaste treatment and expressed as percent remineralization (%Rem). Statistical analyses included paired *t* tests for within-group changes and one-way ANOVA with Tukey’s post hoc tests for between-group comparisons (α = 0.05).

**Results:**

All groups exhibited significant (paired *t* test, *p* < 0.001) increase in SMH from baseline, indicating remineralization. Combining CaP and HAP achieved significantly (ANOVA/Tukey’s, *p* < 0.001) greater %Rem (89.7 ± 3.3) when compared with CaP alone (75.4 ± 5.5), HAP alone (62.4 ± 4.8), or NaF alone (60.3 ± 7.8). While %Rem achieved with CaP alone was significantly (*p* < 0.001) greater than that of HAP and NaF, there was no significant difference in %Rem between HAP and NaF.

**Conclusion:**

This in vitro study demonstrates that CaP alone, and in combination with HAP, increases enamel surface microhardness, confirming that CaP-based formulations are efficient strategies for the remineralization of early caries lesions.

## Introduction

Toothpastes are an essential part of caries prevention [1]. Modern toothpaste formulations comprise several functional classes of ingredients, including cleaning agents, humectants, surfactants, thickeners, and antimicrobial agents [1]. Over the past decades, toothpaste formulations have continuously improved [2]. For example, cleaning agents have become less abrasive while retaining their cleaning efficacy [3]. Furthermore, considerable effort has been invested in developing multifunctional toothpastes (e.g., for gum health, whitening, or relief from dentin hypersensitivity), leading to increasingly complex formulations [1]. For caries prevention, not only single actives but the entire formulation is relevant, in particular remineralizing agents together with plaque-reducing components such as antibacterial agents, cleaning agents, and surfactants [4].

Biomimetic approaches have gained increasing importance across many technological fields [5], and also play a key role in oral care, where they are regarded as safe and promising options [6]. Since human enamel consists of approximately 97% hydroxyapatite (HAP) [7], synthetic particulate HAP is used as an active ingredient in toothpastes [8, 9]. HAP has been shown to be effective in caries prevention [8], remineralization [10], relief of dentin hypersensitivity [11], erosion protection [12], whitening [13], improvement of periodontal health [14], biofilm control [15], and as a special oral care agent for patients with molar incisor hypomineralization (MIH) [16].

Many biomimetic approaches focus on mimicking mature enamel crystallites, i.e., the hydroxyapatite-based mineral phase in its final state [6]. Additional inspiration for improving oral care formulations may be gained by considering enamel formation during amelogenesis. Calcium ions are crucial during amelogenesis because they react with phosphate ions to form HAP [17]. Moreover, saliva naturally contains calcium ions and thus contributes to the continuous remineralization of tooth surfaces [18]. Accordingly, the use of soluble calcium salts represents a promising strategy in oral care.

**Fig. 1:**
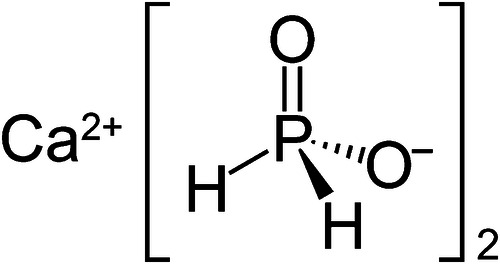
Molecular structure of calcium hypophosphite (CaP).

Calcium hypophosphite (CaP), also known as calcium phosphinate, is a new active ingredient in oral care (Fig. 1). It has properties that make it a particularly promising candidate for remineralization: It exhibits a high water solubility (154 g/L) [19], providing calcium ions readily. In addition, CaP is considered safe and is already used as a calcium source in food additives and dietary supplements [20, 21].

For caries prevention, effective remineralization is essential to counteract demineralization. Fluoride is known to be the standard agent to increase the hardness of previously demineralized enamel [22, 23]. Out of various analytical methods, surface microhardness (SMH) testing is a well-established method to assess remineralization efficacy of toothpastes [24].

The aim of the present in vitro pH-cycling study, designed to imitate the natural fluctuation between de- and remineralization in the oral cavity during the day, was to evaluate the remineralization efficacy of CaP compared to HAP and sodium fluoride using SMH measurements.

Furthermore, the study investigated whether CaP could enhance the caries-related remineralization efficacy of HAP when both actives are combined. Our null hypothesis (H0) was that the group means for the experimental products are equal with respect to percentage remineralization (%Rem) following 14 days of treatment (no significant difference). The alternative hypothesis (H1) was that the group means for the experimental products are not equal with respect to %Rem following 14 days of treatment (significant difference).

## Materials and methods

### Sample preparation

Following approval by the Institutional Animal Care and Use Committee (TR202500000010), freshly extracted bovine teeth were obtained (Animal Technologies, Tyler, TX, USA; Lot #8-210519). The teeth were cleaned using a fine pumice slurry applied with an electric toothbrush (Braun Oral-B Plaque Remover 3D) and subsequently screened by transillumination [25, 26]. Teeth exhibiting cracks, hypo-mineralization, white spot lesions, or other structural malformations were excluded. Eligible specimens were stored in 0.1% thymol solution until use [25, 26].

Using a water-cooled diamond wire saw (WELL [Walter Ebner Le Locle] Diamond Wire Saws SA, 2400 Le Locle, Switzerland), a total of 120 tooth blocks (approximately 3 mm × 3 mm × 1.5 mm) were prepared from the labial surface of the crown. The enamel surface and the base of each block were polished to obtain flat, plane-parallel surfaces suitable for surface microhardness (SMH) measurements, using adhesive-backed lapping films with decreasing grit sizes (30 µm to 1 µm) in a MultiPrep™ Precision Polishing machine (Allied High Tech, Cerritos, CA, USA). Subsequently, all surfaces of each block were coated with two layers of acid-resistant nail varnish (Revlon Consumer Products LLC, New York, NY, USA), except for the buccal enamel surface, which was exposed to acid challenge for the induction of initial caries [27].

### Creation of initial caries lesions

Initial caries lesions were created on the buccal surface of each tooth block by subjecting the specimens to a 4-day demineralization process using a validated microbial caries model [28, 29]. Glycerol stock cultures of *Streptococcus mutans* (NCTC 10449, ATCC, Manassas, VA, USA) and *Lactobacillus casei* (NCIB 8820, ATCC, Manassas, VA, USA) were revived separately. A mixed bacterial suspension was then prepared in phosphate-buffered saline (PBS) and standardized spectrophotometrically (Spectronic 20; Bausch & Lomb, Rochester, NY, USA) to approximately 1 × 10⁷ cells/mL (optical density = 0.25 at 520 nm).

Biofilm formation of the mixed bacterial culture on the tooth samples was carried out using the method described by Amaechi et al. [28]. Briefly, each specimen was placed in an individual well of a 24-well microtiter plate containing pasteurized human whole saliva and incubated at 37 °C in an atmosphere of 5% CO₂ for 30 minutes to allow for acquired salivary pellicle formation. Following pellicle formation, the samples were rinsed with PBS and transferred to new 24-well microtiter plates containing Todd Hewitt broth (THB) inoculated with the mixed *Streptococcus mutans* and *Lactobacillus casei* suspension (1 × 10⁶ cells/mL).

The specimens were then incubated at 37 °C in 5% CO₂ for 24 h to permit bacterial adhesion (adhesion phase). From day 2 onward, the samples were incubated in bacteria-free THB under the same conditions for an additional 3 days. This culture system produces a natural cariogenic biofilm that undergoes daily fasting and feasting cycles, simulating oral conditions, by alternating exposure to a 10% sucrose solution (6 min, three times daily) and growth medium for the remainder of the day. The metabolic activity of *Streptococcus mutans* and *Lactobacillus casei* during the demineralization process was monitored daily by measuring pH changes immediately before and after exposure to the 10% sucrose solution. Plaque pH was also assessed during non-feeding periods to confirm maintenance of neutrality under CO₂ conditions.

### Measurement of the baseline surface microhardness of initial caries lesion

Following the creation of early caries lesions, the baseline surface microhardness (SMH_b_) of each lesion was determined using a microhardness tester (Tukon 2100; Wilson–Instron, Norwood, MA, USA) as described in our previous publication [30]. Each enamel specimen was securely mounted on a 1-inch square acrylic block using sticky wax to ensure stability during testing and then positioned on the stage of the microhardness tester. Surface microhardness measurements were performed using a Vickers diamond indenter. Five baseline indentations were made at the central exposed area of each flattened and polished enamel specimen, with indentations spaced 100 µm apart to prevent interaction between adjacent impressions. A load of 50 g was applied for a dwell time of 15 seconds for each indentation. The SMH values were determined by measuring the lengths of the indentations using the Wilson 2100–Wolpert Image Analysis Software (version 3.5.032; Buehler, Lake Bluff, IL, USA). The five measurements obtained from each specimen were averaged to generate a mean baseline surface microhardness value for each initial caries lesion. Only bovine enamel blocks that demonstrated a minimum of 40% demineralization were selected for the daily remineralization treatment to ensure well-defined and comparable initial lesions.

### Test toothpastes

The compositions of the experimental toothpastes tested in the present study are summarized in Table 1. For the experiments, the same toothpaste base was used for all four toothpastes, differing only in the active ingredients. The toothpastes were provided by the sponsor in a blind format, with identical tubes differentiated only by a random code. Unblinding was performed upon completion of the study.

**Table 1:** Compositions of the four experimental toothpastes tested in the present study.

Toothpastes	Ingredients list
Toothpaste with 1% calcium hypophosphite (calcium phosphinate)	Aqua, Hydrated Silica, Glycerin, Sorbitol, Aroma, Sodium Myristoyl Sarcosinate, Tetrapotassium Pyrophosphate, Silica, Calcium Phosphinate, Cellulose gum, Menthol, Zinc PCA, Sodium Methyl Cocoyl Taurate, Sodium Saccharin, Phenoxyethanol, Anethole, Sodium Hydroxide, Benzyl alcohol, Propylparaben, Methylparaben, Beta-Caryophyllene, Terpineol, Limonene, Citric acid, Pinene, Sodium Benzoate.
Toothpaste with 20% microcrystalline hydroxyapatite	Aqua, Hydroxyapatite, Hydrated Silica, Glycerin, Sorbitol, Aroma, Sodium Myristoyl Sarcosinate, Tetrapotassium Pyrophosphate, Silica, Cellulose Gum, Menthol, Zinc PCA, Sodium Methyl Cocoyl Taurate, Sodium Saccharin, Phenoxyethanol, Anethole, Benzyl Alcohol, Propylparaben, Methylparaben, Beta-Caryophyllene, Terpineol, Limonene, Citric Acid, Pinene, Sodium Benzoate.
Toothpaste with 20% microcrystalline hydroxyapatite and 1% calcium hypophosphite (calcium phosphinate)	Aqua, Hydroxyapatite, Hydrated Silica, Glycerin, Sorbitol, Aroma, Sodium Myristoyl Sarcosinate, Tetrapotassium Pyrophosphate, Silica, Calcium Phosphinate, Cellulose Gum, Menthol, Zinc PCA, Sodium Methyl Cocoyl Taurate, Sodium Saccharin, Phenoxyethanol, Anethole, Benzyl Alcohol, Propylparaben, Sodium Hydroxide, Methylparaben, Beta-Caryophyllene, Terpineol, Limonene, Citric Acid, Pinene, Sodium Benzoate.
Toothpaste with sodium fluoride (1450 ppm fluoride)	Aqua, Hydrated Silica, Glycerin, Sorbitol, Aroma, Sodium Myristoyl Sarcosinate, Tetrapotassium Pyrophosphate, Silica, Cellulose Gum, Menthol, Zinc PCA, Sodium Methyl Cocoyl Taurate, Sodium Fluoride, Sodium Saccharin, Phenoxyethanol, Anethole, Benzyl Alcohol, Propylparaben, Sodium Hydroxide, Methylparaben, Beta-Caryophyllene, Terpineol, Limonene, Citric Acid, Pinene, Sodium Benzoate.

### Power analysis and sample size calculation

To establish the required sample size, G*Power statistical software 3.1.9.7 for Window (Heinrich-Heine-Universität, Düsseldorf, Germany) was used to perform a power analysis. Based on the effect sizes derived from previous studies [30–32] were used, with a confidence level of 95% (α = 0.05) and a statistical power of 80% (1 − β = 0.80) set as the analytical parameters. The results of the analysis demonstrated that a sample size of 30 specimens per group was adequate to detect a statistically significant difference among groups.

### Specimen allocation

Based on their SMH_b_ values, the 120 lesion-bearing enamel blocks were stratified into four treatment groups (*N* = 30 per group), as shown in Table 1. Allocation of the blocks was performed to ensure that there was no statistically significant difference among the mean SMH_b_ values of the groups at baseline. For treatment, the 30 blocks in each group were embedded using dental heavy-duty putty into oblong grooves carved within a cylindrical acrylic rod. This rod was affixed to the inner surface of the lid of a 250 mL treatment tube, allowing the specimens to remain suspended and fully immersed in the treatment solutions during the experimental procedures.

### Remineralization treatment procedure

All four groups were subjected to a standardized pH-cycling regimen consisting of alternating demineralization and remineralization phases, designed to simulate key oral environmental conditions and to evaluate the remineralization potential of each toothpaste formulation. Artificial saliva (AS) was used as the simulated oral fluid for specimen storage and remineralization periods. The artificial saliva was composed of MgCl₂·6H₂O (0.03 g/L), K₂HPO₄ (0.804 g/L), KH₂PO₄ (0.326 g/L), KCl (0.625 g/L), calcium lactate (0.385 g/L), and methyl-4-hydroxybenzoate (2 g/L), with the pH adjusted to 7.2 using KOH [26, 30]. An acidified buffer solution consisting of 2.2 mmol/L KH₂PO₄, 2.2 mmol/L CaCl₂, and 50 mmol/L acetic acid, with the pH raised to 4.5 using KOH, [26, 30] was used as the demineralizing solution (DS) and served as the acidic challenge medium [30].

The daily cyclic treatment regimen (Table 2), as described by Amaechi et al. [30], consisted of a 2-h acidic challenge, three separate 2-min toothpaste treatment periods, and storage in artificial saliva for the remainder of the day. All treatments were carried out with the specimens immersed in the respective treatment media while placed on a laboratory rocker (Labnet Rocker; Stellar Scientific, Baltimore, MD, USA) operating at 350 rpm and housed within a reach-in incubator maintained at 37 °C.

**Table 2:** pH cycling treatment sequence for the experiment.

Daily Events	Treatment
2 minutes	Toothpaste treatment
4 hours	Storage in artifical saliva
2 hours (12 Noon)	Acidic challenge
2 minutes	Toothpaste treatment
4 hours	Storage in artifical saliva
2 minutes	Toothpaste treatment
Till 8:00 A.M. next day	Storage in artifical saliva

For each treatment phase, the specimens in each group were immersed in 200 mL of the appropriate treatment medium (acidic challenge solution, artificial saliva, or toothpaste slurry) within a 250 mL treatment tube. The pH of each treatment medium was measured once daily prior to use. Following exposure to each medium, the specimens were rinsed thoroughly with running deionized water and gently dried with a paper towel before immersion in the subsequent medium. This daily treatment cycle was repeated continuously for a total duration of 14 days.

### Post-remineralization surface microhardness of initial caries lesions

Following completion of the remineralization treatment regimen, the SMH of the remineralized lesions (SMH_r_) was measured using the same microhardness testing protocol described for baseline assessment. Each specimen was repositioned on the microhardness tester, and five post-remineralization indentations were made using a Vickers diamond indenter under identical testing conditions. The post-remineralization indentations were placed 100 µm to the right of the corresponding baseline indentations to ensure that measurements were obtained from a previously untested area of the lesion surface while maintaining comparable lesion characteristics. The lengths of the indentations were measured using the same image analysis software, and the five values obtained for each specimen were averaged to generate a mean SMH_r_ value for each enamel block. At this stage of the experiment, both the post-demineralization surface microhardness (SMH_b_) and the post-remineralization surface microhardness (SMH_r_) values were available for each lesion, allowing for assessment of changes in SMH associated with the remineralization treatment.

### Efficacy measurement

The primary efficacy was determined by the percentage change in SMH (%ΔSMH) relative to baseline, which is a measure of the percentage remineralization (%Rem) achieved with each toothpaste following the 14 days of treatment. This was used for the between-group comparisons and was calculated as:

$$\% {{{\rm{Change}}}}\; {{{\rm{in}}}}\; {{{\rm{SMH}}}}( \% \Delta {{{\rm{SMH}}}})=[({{{\rm{SMHr}}}}-{{{\rm{SMH}}}}_{{{\rm{b}}}})/{{{\rm{SMH}}}}_{{{\rm{b}}}}]\times 100 \%$$

### Statistical analysis

Within groups, SMH_b_ and SMH_r_ were compared using paired t-tests to test for significant treatment-related microhardness gains (remineralization). These treatment comparisons were performed through two statistical analyses using SPSS v28, with a *p*value of ≤0.05 considered statistically significant. The first analysis was the within groups that compared the mean Vickers Hardness Number (VHN) between demineralized and remineralized lesions for each product to determine whether the difference was significantly greater than zero, which indicates remineralization. A one-sided paired samples *t*-test was used to evaluate this research question. Prior to conducting the *t*-test, assumptions were assessed, including the normality of the difference scores, to ensure the appropriateness of the test. The second analysis tested the research hypotheses by comparing the percentage remineralization achieved with the four products. Given that more than two groups were being compared, a one-way ANOVA was conducted. A statistically significant omnibus F-test (*p* ≤ 0.05) indicated overall group differences, prompting post hoc tests to identify which specific group means differed significantly from one another.

### Ethics declaration

Following approval by the Institutional Animal Care and Use Committee (TR202500000010), freshly extracted bovine teeth were used for this study (Animal Technologies, Tyler, TX, USA; Lot #8-210519).

## Results

**Fig. 2:**
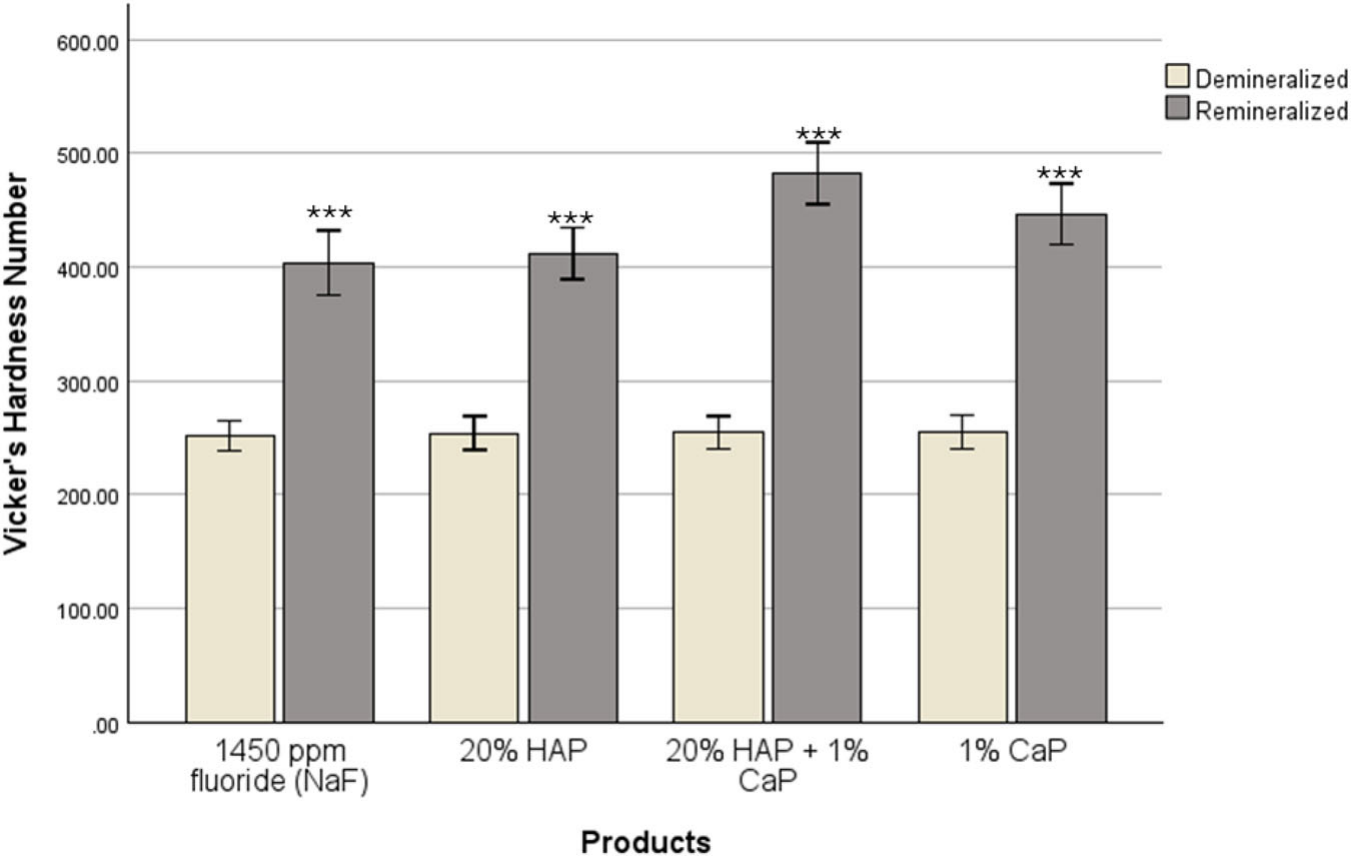
Pre-/post-treatment microhardness. Within group comparison of pre-treatment and post-treatment mean microhardness. ***Indicates statistically significant difference between pre-treatment and post-treatment (*p* < 0.001).

Prior to remineralization procedure, a one-way ANOVA confirmed there were no statistically significant differences (F(3,56) = 0.788, *p *= 0.506) in the mean SMH among the treatment groups. Following remineralization, all groups exhibited statistically significant (paired *t* test, *p* < 0.001) increase in SMH from baseline, indicating remineralization (Fig. 2).

**Fig. 3:**
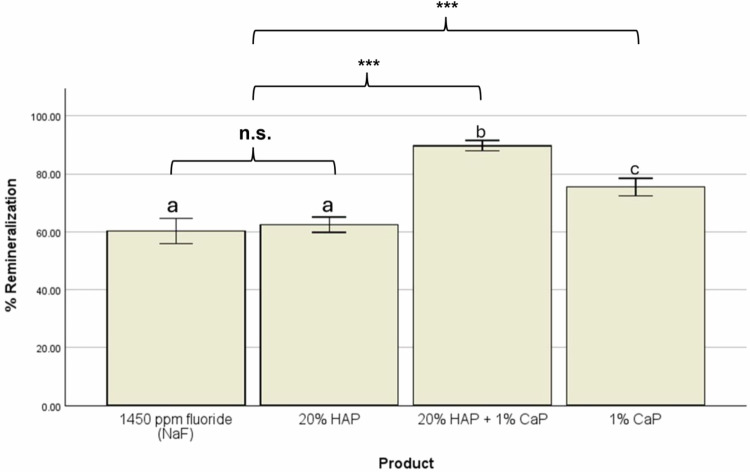
Remineralization with toothpastes. Comparing the treatment groups based on the percentage remineralization achieved with each treatment product. Groups with similar letters (**a**–**c**) are not statistically significantly different, while different letters indicate statistically significant difference (*p* < 0.001).

A one-way ANOVA showed a statistically significant difference in percentage remineralization (%ΔSMH) among the four groups (F(3,56) = 88.77, *p* < 0.001) at the alpha level of 0.05. This was followed by post hoc pairwise comparisons of groups using the Tukey test to identify the specific group means that significantly differed from each other. This test indicated that combining 20% HAP and 1% CaP achieved significantly (*p* < 0.001) greater percentage remineralization (%Rem) than 1450 ppm fluoride (NaF), 20% HAP, and 1% CaP (Fig. 3, Table 3). While the %Rem achieved with 1% CaP was significantly (*p* < 0.001) greater than that of 20% HAP and NaF, there was no significant difference in %Rem between 20% HAP and NaF (Fig. 3, Table 3).

**Table 3:** Tukey HSD multiple comparisons of the products based on their achieved mean percentage remineralization (dependent variable).

(I) Product	(J) Product	Mean Difference (I-J)	Std. Error	Sig.	95% Confidence Interval	
					Lower Bound	Upper Bound
1450 ppm fluoride (NaF)	20% HAP	–2.11442	2.03886	0.729	–7.5131	3.2843
	20% HAP + 1% CaP	–29.39350^*^	2.03886	<0.001	–34.7922	–23.9948
	1% CaP	–15.12020^*^	2.03886	<0.001	–20.5189	–9.7215
20% HAP	1450 ppm fluoride	2.11442	2.03886	0.729	–3.2843	7.5131
	20% HAP + 1% CaP	–27.27908^*^	2.03886	<0.001	–32.6778	–21.8804
	1% CaP	–13.00579^*^	2.03886	<0.001	–18.4045	–7.6071
20% HAP + 1% CaP	1450 ppm fluoride	29.39350^*^	2.03886	<0.001	23.9948	34.7922
	20% HAP	27.27908^*^	2.03886	<0.001	21.8804	32.6778
	1% CaP	14.27329^*^	2.03886	<0.001	8.8746	19.6720
1% CaP	1450 ppm fluoride	15.12020^*^	2.03886	<0.001	9.7215	20.5189
	20% HAP	13.00579^*^	2.03886	<0.001	7.6071	18.4045
	20% HAP + 1% CaP	–14.27329^*^	2.03886	<0.001	–19.6720	–8.8746

*The mean difference is significant at the 0.05 level.

## Discussion

Inspired by natural processes, such as mineral phase formation during amelogenesis [17], and enamel remineralization from saliva [18], the use of water-soluble calcium compounds represents a promising approach for enamel remineralization. In this context, CaP is an interesting active ingredient for use in toothpastes because it is highly water-soluble [19], and thus, it provides calcium ions.

The present study is the first to evaluate the caries-remineralization efficacy of CaP using SMH testing. There are several main findings: CaP alone was demonstrated to be an efficient active ingredient to increase SMH of previously caries-demineralized enamel. It achieved significantly greater %Rem compared to HAP and sodium fluoride. The highest %Rem was observed when CaP and HAP were combined in one toothpaste formulation (Fig. 3, Table 3). This shows that, in particular, the combination of CaP and HAP is a promising approach for advanced oral care remineralizing formulations.

Hydroxyapatite is known to fill micropores in enamel, forming mineral bridges and a protective surface layer on enamel [12, 33]. It can also release calcium ions, but only under acidic conditions [34]. In contrast, CaP is readily soluble in water (also at neutral pH) [19]. In a previous study, it was shown that CaP reacts with artificial saliva to form mineral precipitates consisting of HAP [35]. It is likely that these precipitates contributed to the observed increase in SMH of caries-demineralized enamel in the present study. The combined system of CaP and HAP shows a pronounced synergistic effect on enamel remineralization (Fig. 3, Table 3). A plausible mechanism could be that HAP acts as a crystal nucleus, attracting calcium ions (from CaP) and phosphate ions (from saliva), thereby promoting mineral deposition [18]. Thus, two complementary modes of action – surface remineralization by HAP, and ion supply with subsequent HAP precipitation from CaP – act together to enhance enamel remineralization.

The remineralization efficacy of toothpaste with HAP or with sodium fluoride observed in the present study is in line with published data: The remineralization effect of a HAP toothpaste, measured by SMH, has been demonstrated in a previous in vitro study [36]. In that study, a toothpaste containing 10% HAP led to a hardening of artificially caries-demineralized enamel that was not significantly different from that achieved with a tricalcium phosphate toothpaste or a toothpaste with fluoride. The enamel hardening effect of fluoride, as described in other studies [22, 23], has been confirmed in the present study.

A previous study has demonstrated that HAP can also remineralize deeper enamel lesions [31]. In contact with fluoride ions, calcium ions from enamel directly react to form the mineral precipitate calcium fluoride (CaF₂) [37]. This provides a plausible mechanism, though not physicochemically examined in the present study, whereby the observed increase in hardness in the fluoride group is driven by an increased mineral density at the enamel surface due to CaF₂ formation. As a mineral, however, CaF₂ (K_sp_ ≈ 3.6 × 10⁻¹¹ [38]) is substantially more soluble than HAP (K_sp_ ≈ 1 × 10⁻⁵² [39]), which may reduce its protective efficacy against acidic challenges compared with HAP.

This study has several methodological strengths. A microbiological caries model that induces caries-like lesions comparable to those observed in situ studies, a frequently used method to quantitatively assess remineralization efficacy, was used to create the initial caries in this study. Moreover, a well-established pH-cycling model was applied that mimics the natural alternation of demineralization and remineralization phases during the day. Although a cyclic demineralization-remineralization procedure was used, an important limitation is that the full complexity of the oral cavity (e.g., proteins, salivary flow, pellicle formation) was not considered in this in vitro setting. Nevertheless, the present in vitro findings provide a strong rationale for the further clinical evaluation of CaP-containing toothpastes. Since the present study evaluated the remineralization efficacy of 1% CaP, and calcium ions are essential for enamel remineralization, future in vitro studies could also investigate higher CaP concentrations.

## Conclusions

This surface microhardness study demonstrates that combining calcium hypophosphite with hydroxyapatite results in significantly higher %Rem than calcium hypophosphite alone, hydroxyapatite alone, or sodium fluoride alone. Calcium hypophosphite alone achieved significantly greater %Rem than hydroxyapatite alone or sodium fluoride alone, while no significant difference was observed between hydroxyapatite and sodium fluoride. Calcium hypophosphite readily provides calcium ions, which reacts with phosphate ions in saliva to form calcium phosphate precipitates containing hydroxyapatite. Overall, calcium hypophosphite alone, and especially in combination with hydroxyapatite, represents an efficient agent for enamel caries remineralization.
